# The neonatal and maternal nasal microbiomes are compositionally diverse and functionally distinct within 24 hours of birth

**DOI:** 10.3389/fcimb.2026.1922369

**Published:** 2026-09-01

**Authors:** Sheridan B. Wagner, Katelyn E. Keen, Isaac Cinco, Mónica Rincón, Nicole E. Marshall, Ilhem Messaoudi

**Affiliations:** 1 Microbiology, Immunology and Molecular Genetics, College of Medicine, University of Kentucky, Lexington, KY, United States; 2 Division of Maternal-Fetal Medicine, Department of Obstetrics and Gynecology, Oregon Health & Science University, Portland, OR, United States

**Keywords:** 16S rRNA gene sequencing, maternal-neonatal dyad, nasal microbiome, neonate, PICRUSt2, respiratory health, vertical transmission

## Abstract

**Background:**

The nasal microbiome provides a first line of defense at the respiratory mucosa, yet its composition immediately after birth and its relationship to the maternal nasal community remain poorly defined.

**Methods:**

We used 16S rRNA gene sequencing to compare anterior nares swabs collected within 24 hours of delivery from mothers (n=25) and newborns (n=34), including 22 maternal–newborn dyads.

**Results:**

The neonatal nasal microbiome was significantly more diverse than the maternal microbiome, with higher Shannon diversity, and the two communities were compositionally distinct by weighted UniFrac analysis. Maternal samples were dominated by *Corynebacterium* and *Dolosigranulum*, genera characteristic of a stable, healthy adult nares, whereas newborn samples were enriched for *Lactobacillus, Burkholderia-Caballeronia-Paraburkholderia, Acinetobacter*, and *Streptococcus*. Predicted functional profiling with PICRUSt2 indicated that, relative to mothers, neonatal communities had greater predicted capacity for degradation of aromatic and xenobiotic compounds, biosynthesis of antimicrobial secondary metabolites (including polyketides, β-lactams, and terpenoids), and lipid metabolism.

**Conclusions:**

Together, these findings show that the newborn nasal cavity is not a sparsely colonized niche but instead harbors a compositionally diverse and functionally versatile microbial community that is distinct from the maternal nares in both membership and predicted metabolic potential. These results identify the perinatal period as a critical window for nasal microbial colonization, with potential implications for the trajectory of infant respiratory health.

## Introduction

The human nasal mucosa houses a complex microbial community that serves as the first line of defense against respiratory infections and impacts the development of the mucosal immune system (Man et al., 2017; Lee et al., 2019) through secretion of metabolites and physically outcompeting potential pathogens (McDonogh, 1986; Kumpitsch et al., 2019; Mukhtar et al., 2026). Healthy adult nasal microbiomes are marked by stable populations of *Corynebacterium* (Actinobacteria), *Staphylococcus* (Firmicutes), *Dolosigranulum*, and *Moraxella* (Proteobacteria) genera (Biswas et al., 2015). Factors such as sex, age, antibiotic use, seasonality, and pollution contribute to the variation in microbe proportions observed between individuals and can serve as predictors of respiratory disease (Kang and Kang, 2021; Ruttimann et al., 2023).

Postnatal microbial colonization of newborn mucosal surfaces is increasingly recognized as a determinant of long-term immune programming and pathogen susceptibility (Backhed et al., 2015; Ferretti et al., 2018). Growing evidence suggests that early colonization of the nasal cavity modulates the risk of asthma and other respiratory diseases in children (Toivonen et al., 2019; Chun et al., 2020; Drigot and Clark, 2024). Accordingly, the stability and functional capacity of early nasal colonization may have lasting consequences for respiratory health in infancy, including susceptibility to acute respiratory infections such as respiratory syncytial virus infection, pneumonia, and bronchiolitis, which remain major causes of hospitalization before five years of age (de Steenhuijsen Piters et al., 2016; Lee et al., 2019; Toivonen et al., 2019; Alba et al., 2021). Longitudinal infant studies have shown that nasal microbial communities fluctuate throughout the first year of life before stabilizing (Peterson et al., 2016; Bogaert et al., 2023). However, the newborn nasal microbiome remains comparatively understudied, particularly during the first day after delivery.

In this study, we used 16S rRNA gene sequencing to characterize the composition and predicted functional profiles of maternal and newborn nasal microbiomes from samples collected within 24 hours of delivery. We aimed to compare the taxonomic composition and diversity of the maternal and neonatal nasal microbiome at birth, evaluate the contribution of mode of delivery to newborn nasal colonization, and characterize predicted functional differences between these two communities using PICRUSt2-based functional profiling.

## Methods

### Cohort description

This study was approved by the Institutional Review Boards at Oregon Health & Science University and the University of Kentucky. Written informed consent for the use of samples in research was obtained from participants at enrollment. Maternal and newborn participants were enrolled at Oregon Health & Science University. Enrolled patients were at least 18 years-of-age and antepartum. Patients with an active hepatitis infection, HIV infection, or history of substance use disorder (non-prescription opiate, stimulant, or alcohol) were excluded from enrollment. Anterior nares swabs were collected from maternal participants (n=25) upon admission for delivery and from newborn participants (n=40) within 24 hours of delivery. Six newborn swabs were removed post-sequencing leaving n=34 for analysis. Of these participants, 22 were paired maternal-newborn dyads (**Table 1**). Nasal swabs were placed in 1x DNA/RNA Shield (Zymo Research) and stored at -80 °C until processing.

**Table 1:** Maternal characteristics of cohorts.

Maternal characteristics of cohorts		Maternal (n=25)	Newborn (n=34)
Maternal Age at Delivery (years)		32.6 ± 2.7	33.1 ± 2.9
Maternal Age at Delivery Range		27 – 37	27 – 40
Pre-Pregnancy BMI		26.5 ± 7.1	26.3 ± 7.0
Pre-Pregnancy BMI Range		19.4 – 41.6	19.4 – 41.6
Gestational Age at Delivery (weeks)		39.0 ± 1.7	39.1 ± 1.4
Fetal Sex % Female		52.0%	58.8%
Mode of Delivery	Vaginal	16 (64%)	24 (70.6%)
	Assisted Vaginal	1 (4%)	0 (0%)
	Scheduled Cesarean	3 (12%)	7 (20.6%)
	Unscheduled Cesarean	5 (20%)	3 (8.8%)
Race	American Indian/Alaskan Native	0 (0%)	1 (2.9%)
	Asian American	1 (4%)	2 (5.9%)
	Black/African American	2 (8%)	2 (5.9%)
	Unknown/ Declined to state	3 (12%)	3 (8.8%)
	White/Caucasian	19 (76%)	26 (76.5%)

No significant difference between maternal and newborn nasal cohort was determined for maternal age at delivery, pre-pregnancy BMI, gestational age at delivery, fetal sex, mode of delivery, or race.

### 16S rRNA amplicon sequencing

Total DNA was extracted from nasal swabs using the DNeasy PowerSoil Pro Kit (Qiagen) using an input volume of 150-600 μL and elution volume of 50 μL. Negative extraction controls were collected alongside each extraction batch. The hypervariable V4 region of the 16S rRNA gene was amplified using the 515F forward primer (Parada) and 806R reverse primer (Apprill) (Apprill et al., 2015; Parada et al., 2016). Technical duplicates were amplified by polymerase chain reaction (PCR) using 12.5 μL GoTaq master mix, 9.5 μL nuclease-free water, 1 μL template DNA, and 1 μL 10 μM primer mix. PCR cycling conditions were 94 °C for 5 min; 35 cycles of 94 °C for 20 s, 50 °C for 20 s, and 72 °C for 30 s; and a final extension at 72 °C for 5 min. Duplicate negative template PCR controls were also amplified. Technical duplicates from the same sample were pooled, run on an agarose gel to confirm selective amplification of the 515F-806R amplicon, and quantified using the Quant-iT PicoGreen dsDNA Assay Kit (Invitrogen). Samples were multiplexed at 300 ng/μL and cleaned using the MinElute 96 UF PCR Purification Kit (Qiagen). Purified multiplexed pools were assessed on a High Sensitivity DNA Bioanalyzer (Agilent) to confirm PCR product quality and sequenced on an Illumina MiSeq.

### 16S rRNA data analysis

16S rRNA sequences were analyzed using the QIIME2 pipeline (Bolyen et al., 2019). Sequences were demultiplexed, and adapter, primer, and linker pad sequences were removed with qiime cutadapt trim-paired. Denoising and quality-control filtering were performed using DADA2 (trim-left-f 10, trim-left-r 10, trunc-len-f 240, trunc-len-r 205, n-threads 60). Sequences were aligned with MAFFT, and a phylogenetic tree was built using FastTree. A taxonomy classifier was trained using the SILVA SSURef_NR99 database (v. 138.2) and used to assign taxonomy to sequence variants (ChuvoChina et al., 2026). Contaminant taxa were identified using the decontam prevalence method on negative extraction and PCR control gene copy numbers separately. Taxa with a score < 0.5 were designated as contaminants and removed from subsequent analyses (**Supplementary Table 1**). Archaea and unassigned reads were removed. Alpha rarefaction (max-depth 10000, min-depth 600) and beta rarefaction (metric braycurtis, clustering-method nj, sampling-depth 4229) were performed to assess the impact of sampling depth on alpha and beta diversity.

To prevent sequencing-depth bias, samples were rarefied to 4229 sequences per sample. QIIME2 was used to calculate observed amplicon sequence variants (ASVs), Shannon diversity, and weighted UniFrac distance. Weighted UniFrac was selected as the primary beta diversity metric because it incorporates both phylogenetic relatedness and relative taxon abundance, which is relevant given the taxonomic composition of the upper respiratory tract microbiome (e.g. closely related *Actinobacter* genera).

### Statistical analysis

Statistical two-group comparisons of observed amplicon sequence variants (ASVs) and Shannon diversity scores were performed using the nonparametric Mann-Whitney test in GraphPad Prism following outlier removal with ROUT (Q = 1). Unpaired comparisons of genus-level percent relative abundances were performed using the nonparametric Mann-Whitney test. Weighted UniFrac distances underwent permutation-based testing of multivariate group dispersions (betadisper/permutest) between maternal and newborn samples to observe inter-individual variability. For paired analyses of matched maternal-newborn dyads, the nonparametric Wilcoxon matched-pairs signed-rank test was used. Principal coordinate analysis (PCoA) was visualized using Tableau. Linear discriminant analysis Effect Size (LEfSe) was performed on genus-level (level 6) taxonomic features percent relative abundances to identify differentially abundant taxa between maternal and newborn samples using an LDA score threshold of ≥ 2.0, for features present at an average relative abundance of greater than 0.5% and a sample prevalence greater than 10% (Segata et al., 2011). Phylogenetic Investigation of Communities by Reconstruction of Unobserved States 2 (PICRUSt2 v2.6.2) was used to predict the functional potential of microbial communities from 16S rRNA amplicon sequencing data alongside the Kyoto Encyclopedia of Genes and Genomes (KEGG) and MetaCyc pathway databases (Caspi et al., 2020; Douglas et al., 2020; Kanehisa et al., 2025). Differential pathway abundance was determined using linear model for differential abundance (LinDA) and the Benjamini-Hochberg false discovery rate correction.

## Results

### Higher diversity in neonatal nasal microbial communities

We performed 16S rRNA gene sequencing on maternal (n=25) and newborn (n=40; prior to rarefied exclusion for analysis) nasal swabs collected within 24 hours of delivery, including 22 paired maternal-newborn dyads, to evaluate nasal microbiome composition and diversity at birth. Biological samples yielded 10,531,524 raw reads (mean 162,023 ± 95,458 reads/sample; range 22,324-402,089). After primer trimming, 9,309,275 reads remained from biological samples. Following DADA2 denoising, 6,209,406 reads were retained from biological samples, comprising 3,615,845 reads from maternal samples and 2,593,561 reads from neonatal samples. After removal of contaminant amplicon sequence variants (ASV) identified via extraction and PCR negative controls using decontam (43 identified contaminant taxa, 2.28% of all features), 5,787,513 reads remained across the 65 biological samples (mean 89,039 ± 66,081 reads/sample post-decontamination; range 669-266,026) (**Supplementary Table 1**).

**Figure 1:**
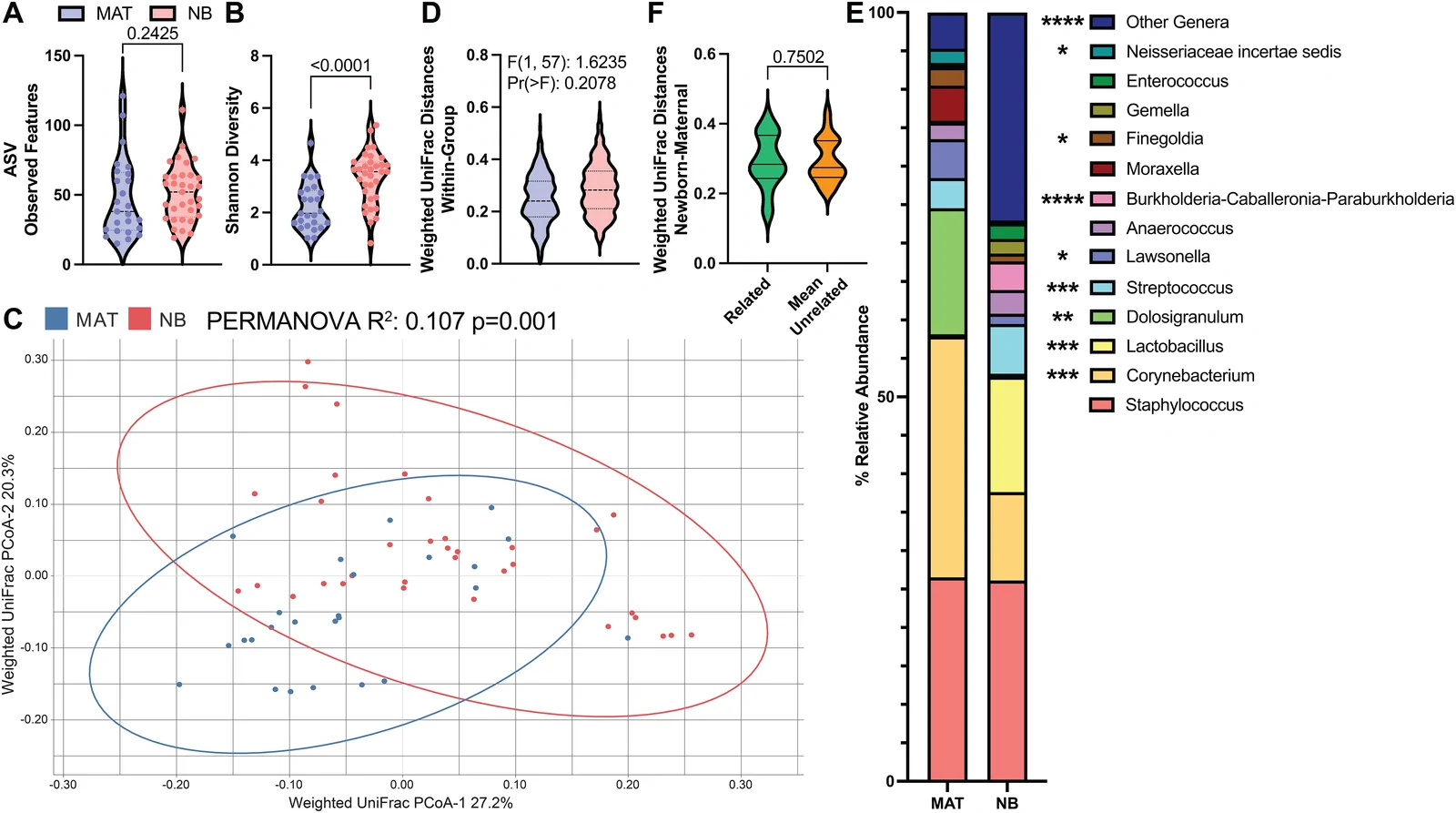
**(A, B)** Violin plots of **(A)** observed amplicon sequence variants (ASVs) and **(B)** Shannon diversity indices. Significance for **(A)** and **(B)** was measured using a Mann-Whitney test following outlier removal with ROUT (Q = 1). **(C)** Principal coordinate analysis (PCoA) of weighted UniFrac distances comparing newborn and maternal nasal microbiota, colored by subject group. The contribution of subject group to observed variance was determined by permutational multivariate analysis of variance (PERMANOVA). Ellipses display 95% confidence intervals. **(D)** Violin plot of weighted UniFrac distances within newborn (NB-NB) and maternal (MAT-MAT) groups. Inter-individual variability in **(D)** was determined by permutation-based testing of multivariate group dispersion. **(E)** Stacked bar plot of genera detected in maternal and newborn samples. Genera present at an average relative abundance <1% were grouped as “Other Genera.”Significance for **(E)** was measured using a Mann-Whitney test. *p < 0.05, **p < 0.01, ***p < 0.001, ****p < 0.0001. **(F)** Violin plots of within-dyad (related) versus mean between-dyad (unrelated) newborn-maternal weighted UniFrac distances compared using a paired Wilcoxon test.

Samples were rarefied to a depth of 4,229 reads to normalize sequencing effort across samples. This depth was selected based on ASV, Shannon diversity, and sample number rare faction curves to maximize observed features and Shannon diversity while minimizing the number of samples excluded (**Supplementary Figure 1A**). Six neonatal samples fell below this threshold and were excluded from downstream diversity analyses, retaining 25 maternal (1187 ASV) and 34 neonatal (1888 ASV) samples and including 22 paired maternal-newborn dyads, to evaluate nasal microbiome composition and diversity at birth (**Table 1**). Among these remaining samples, no significant differences were observed in gestational age at delivery, maternal age at delivery, maternal pre-pregnancy BMI, fetal sex, mode of delivery, or race (**Table 1**). Although maternal and newborn nasal microbial communities did not differ significantly in the number of ASV detected, newborn swabs exhibited higher Shannon diversity (**Figures 1A, B**). Principal coordinate analysis (PCoA) of weighted UniFrac distances further revealed significant compositional separation between maternal and newborn nasal swabs by permutational multivariate analysis of variance (PERMANOVA; R^2^ = 0.107, p = 0.001) (**Figure 1C**). However, the R^2^ value indicated a smaller effect size with only 10.7% of the variance microbial composition being explained by maternal and newborn sample separation (**Figure 1C**). Weighted UniFrac distance was not significantly different across maternal samples than across newborn samples, indicating no significant difference in inter-individual variability (**Figure 1D**). Maternal and newborn nasal microbiomes had similar relative abundance of *Staphylococcus*, whereas maternal communities contained significantly higher proportions of *Corynebacterium*, *Dolosigranulum*, *Lawsonella*, *Finegoldia*, and *Neisseriaceae incertae sedis* (**Figure 1E**). Conversely, *Lactobacillus*, *Streptococcus*, and *Burkholderia-Caballeronia-Paraburkholderia* were significantly enriched in newborn nasal samples (**Figure 1E**). The weighted UniFrac distance between related newborn maternal swabs was compared to the distance between newborn and unrelated maternal swabs in order to assess dyad-specific similarity (**Figure 1F**). Related-dyad distance was not significantly different from unrelated-dyad distance (**Figure 1F**).

### Increased abundance of *Lactobacillus* in newborn nasal cavities

**Figure 2:**
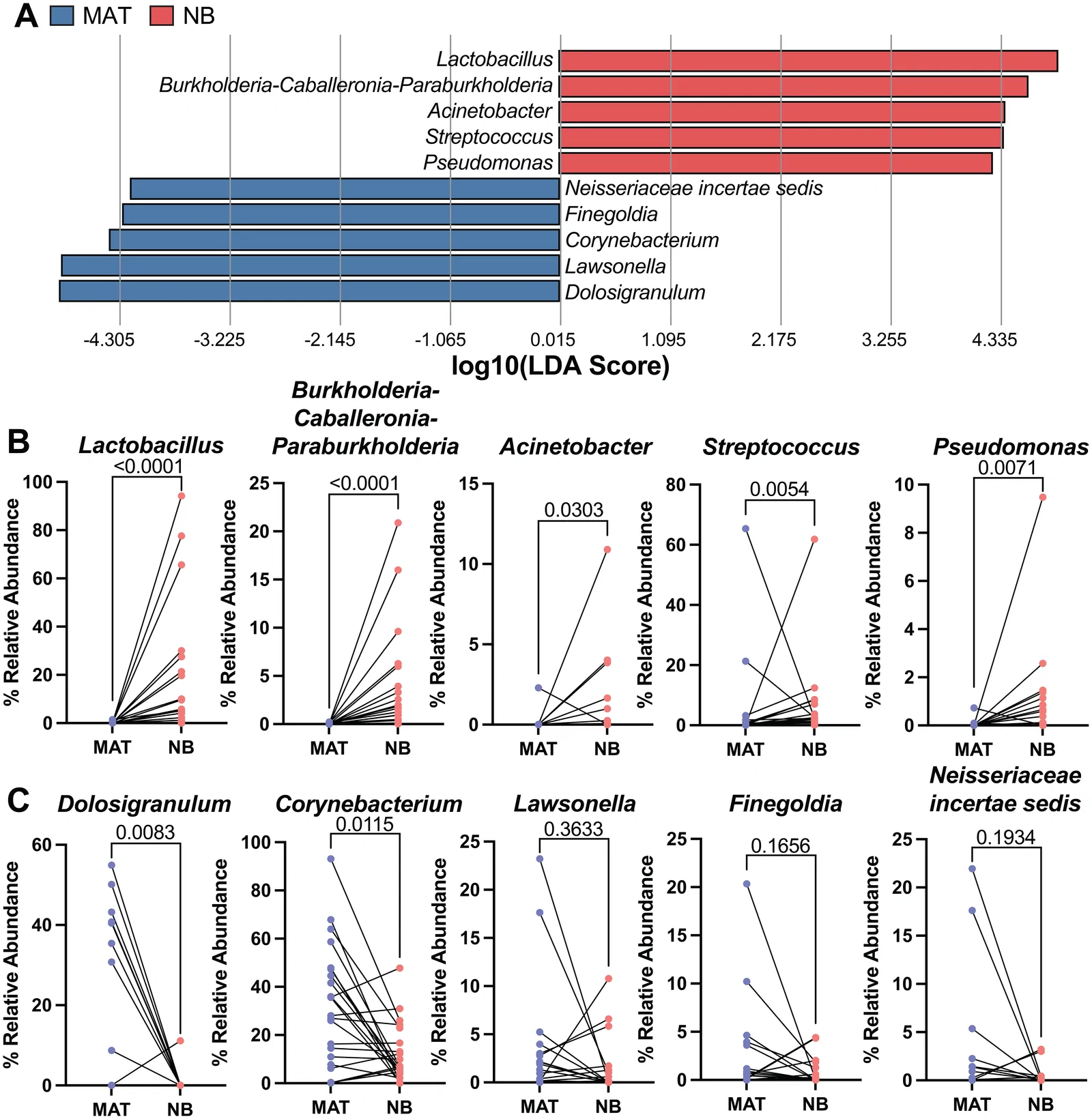
**(A)** Differentially abundant genera between maternal and newborn nasal samples as determined by LEfSe. **(B, C)** Paired dot plots of matched maternal-newborn nasal swab dyads comparing percent relative abundance of selected genera. Lines connect paired maternal and newborn samples from the same dyad. Significance was assessed using the Wilcoxon matched-pairs signed-rank test.

To identify genera driving the differences between maternal and newborn nasal microbial communities, we performed linear discriminant analysis Effect Size (LEfSe) on the relative abundances of selected genera. Comparisons across maternal (n=25) and newborn (n=34) samples revealed significant percent abundance differences for each of these genera (**Supplementary Figure 1B**). Specifically, newborn microbiomes were enriched for *Lactobacillus*, *Burkholderia-Caballeronia-Paraburkholderia*, *Acinetobacter*, *Streptococcus*, and *Pseudomonas* whereas maternal nasal microbiomes were enriched for *Dolosigranulum*, *Lawsonella*, *Corynebacterium*, *Finegoldia*, and *incertae sedis* of the *Neisseriaceae* family (**Figure 2A**). We next performed pairwise comparisons of the 22 matched maternal and newborn nasal swabs (**Figure 2B**). Consistent with the full-cohort analysis, newborn swabs had significantly higher relative abundance of *Lactobacillus*, *Burkholderia-Caballeronia-Paraburkholderia*, *Acinetobacter*, *Streptococcus*, and *Pseudomonas* (**Figure 2B**). *Lactobacillus* abundance varied widely across newborn samples, ranging from 0% to 94%, while paired maternal swabs generally contained approximately 1% or less (**Figure 2B**). The relative abundance of both *Corynebacterium* and *Dolosigranulum* remained significantly higher in paired maternal samples (**Figure 2C**). *Lawsonella*, *Finegoldia*, and *Neisseriaceae incertae sedis* lacked statistical significance between paired newborn and maternal swabs (**Figure 2C**).

**Figure 3:**
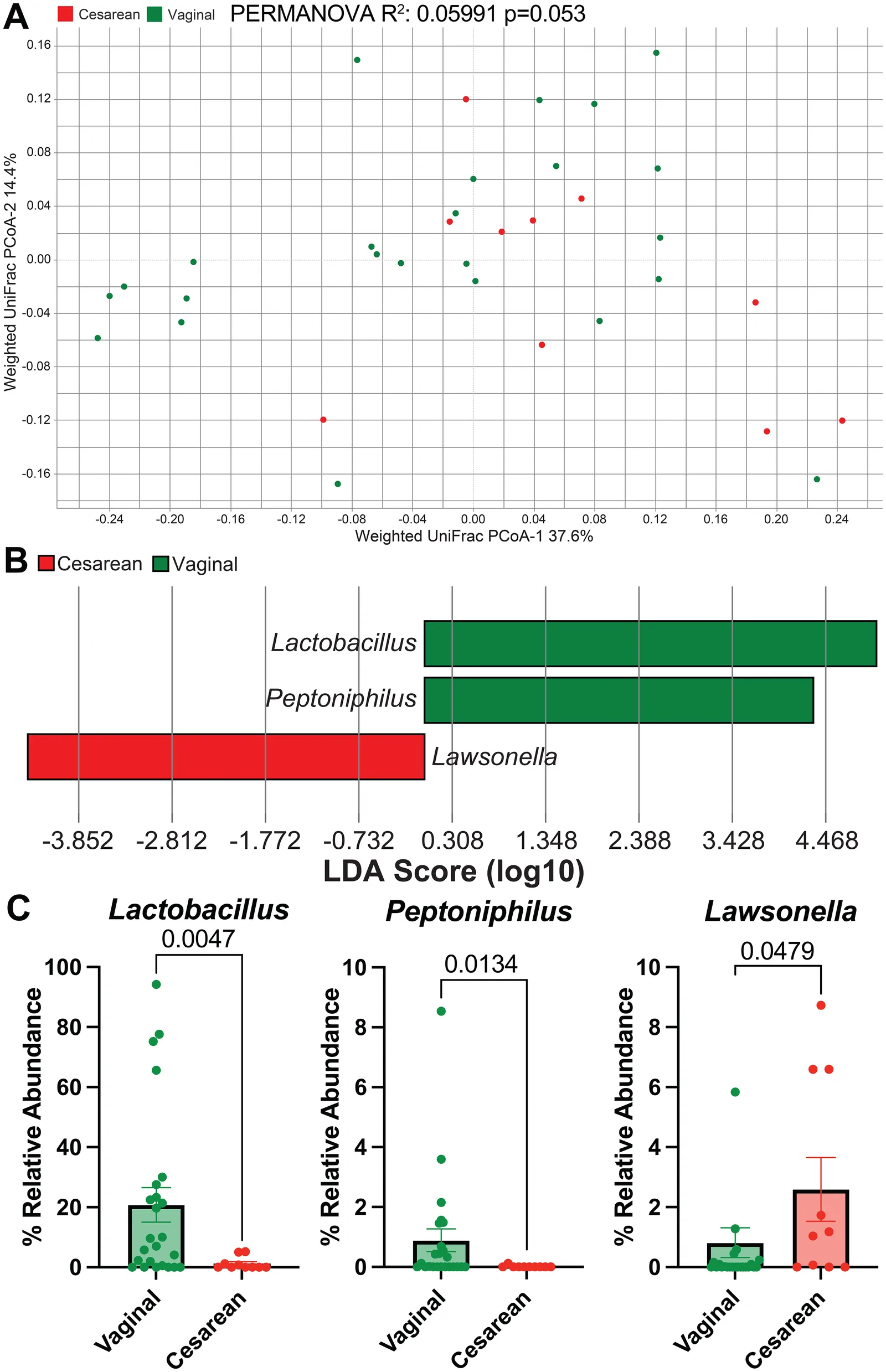
**(A)** Principal coordinate analysis (PCoA) of weighted UniFrac distances comparing the nasal microbiota of newborn delivery by cesarean and vaginally. The contribution of subject group to observed variance was determined by permutational multivariate analysis of variance (PERMANOVA). **(B)** Differentially abundant genera driven by delivery mode in newborn nasal samples as determined by LEfSe. **(C)** Percent relative abundance of selected genera between delivery modes. Significance was assessed using a Mann-Whitney test.

Give the prevalence of *Lactobacillus* within the vaginal microbiome and the wide range of *Lactobacillus* relative abundance within the newborn samples, we assessed the impact of the mode of delivery on the composition of the newborn microbiome. A PCoA of weighted UniFrac distances revealed significant compositional separation between the nasal microbiome of newborns delivered vaginally and cesarean by permutational multivariate analysis of variance (PERMANOVA; R2 = 0.05991, p = 0.053) (**Figure 3A**). We additionally performed a PCoA comparing newborn weighted UniFrac distances separated by fetal sex which revealed no significant difference between female and male samples (**Supplementary Figure 1C**). To identify genera driving the differences with mode of delivery in newborn nasal microbial communities, we performed linear discriminant analysis Effect Size (LEfSe) on the relative abundances of selected genera (**Figure 3B**). Nasal microbiomes of vaginally delivered newborns were enriched for *Lactobacillus* and *Peptoniphilus* while nasal microbiome of newborns delivered by cesarean were enriched for *Lawsonella* (**Figures 3B, C**).

### Increased aromatic compound degradation, antimicrobial biosynthesis, and lipid metabolism predicted in newborn nasal microbiome

**Figure 4:**
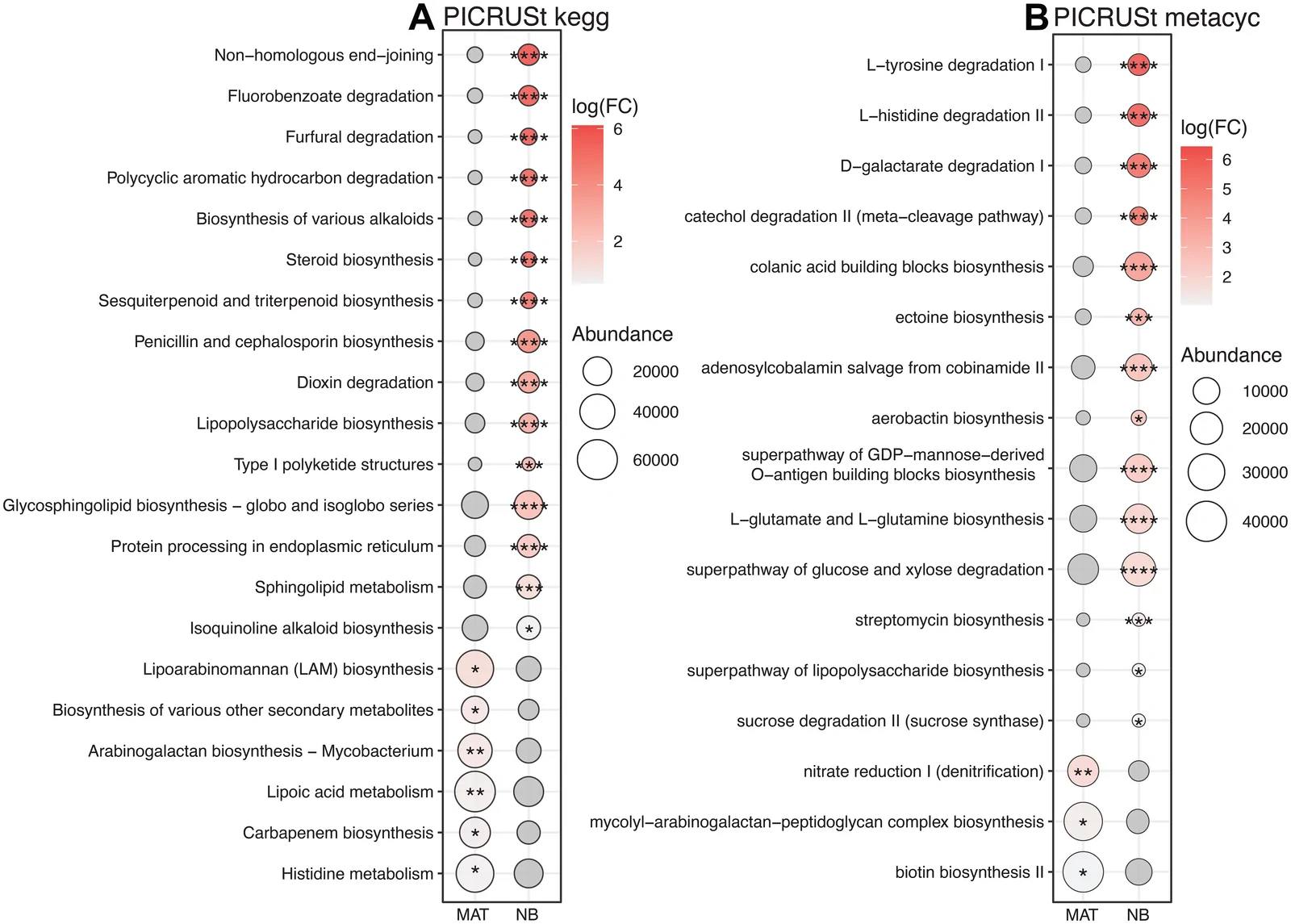
**(A, B)** Bubble plots showing selected differentially abundant predicted metabolic pathways from the **(A)** KEGG and **(B)** MetaCyc databases identified by PICRUSt2 analysis. Bubble size represents predicted relative pathway abundance, and color represents log fold-change between groups. Predicted functional abundances were tested for differential abundance with LinDA and adjusted for multiple testing using the Benjamini-Hochberg false discovery rate procedure. *p < 0.05, **p < 0.01, ***p < 0.001, ****p < 0.0001.

We performed Phylogenetic Investigation of Communities by Reconstruction of Unobserved States 2 (PICRUSt2) analysis using both the Kyoto Encyclopedia of Genes and Genomes (KEGG) and MetaCyc databases to predict functional and metabolic profiles of the newborn and maternal nasal microbiomes (**Figure 4**). Newborn swabs showed greater predicted abundance of pathways involved in xenobiotic and aromatic compound degradation, including polycyclic aromatic hydrocarbon degradation, fluorobenzoate degradation, furfural degradation, and dioxin degradation (**Figure 4A**). The newborn nasal community also showed higher predicted abundance of pathways related to antimicrobial and bioactive product synthesis, including alkaloid biosynthesis, sesquiterpenoid and triterpenoid biosynthesis, penicillin and cephalosporin biosynthesis, type I polyketide structures, and isoquinoline alkaloid biosynthesis (**Figure 4A**). In addition, lipid metabolism pathways, including steroid biosynthesis, glycosphingolipid biosynthesis, lipopolysaccharide biosynthesis, and sphingolipid metabolism, were predicted to be enriched in newborn nasal swabs (**Figure 4A**). Non-homologous end joining and protein processing in the endoplasmic reticulum were also predicted to be higher in newborn samples (**Figure 4A**). Functional pathways predicted to be higher in maternal samples included arabinogalactan biosynthesis, lipoarabinomannan biosynthesis, lipoic acid metabolism, and carbapenem biosynthesis (**Figure 4A**).

Prominent pathways from the MetaCyc database predicted to be increased in the newborn nasal microbiome were involved in carbohydrate metabolism (D-galactarate degradation I, sucrose degradation II, superpathway of GDP-mannose-derived O-antigen building blocks biosynthesis, and superpathway of glucose and xylose degradation) and amino acid metabolism (L-histidine degradation II, L-tyrosine degradation I, L-glutamate and L-glutamine biosynthesis) (**Figure 4B**). Other notable pathways enriched in newborn samples included biosynthesis of colonic acid building blocks, ectoine, aerobactin, streptomycin, and lipopolysaccharide (**Figure 4B**). Pathways enriched in the maternal nasal microbiome included nitrate reduction I, mycolyl-arabinogalactan-peptidoglycan complex biosynthesis, and biotin biosynthesis II (**Figure 4B**).

## Discussion

The nasal microbiome is increasingly recognized as a determinant of early respiratory health, yet its composition in the first hours of life remains poorly defined. Here, by profiling maternal and newborn nasal swabs collected within 24 hours of delivery, we show that the neonatal nasal cavity already harbors a compositionally diverse and functionally versatile microbial community distinct from the maternal nares. These observations are consistent with recent reports of a broad assemblage of low-abundance taxa present in the nasal microbiome at birth (Quinn et al., 2025) that subsequently shifts over the first year of life as the community stabilizes (Accorsi et al., 2020). Longitudinal studies indicate that maternal influence on nasal composition accrues over the first months of life, with the maternal nares serving as a principal source of colonizing bacteria (Accorsi et al., 2020; Quinn et al., 2025). The correspondingly lower diversity and tighter within-group UniFrac distances we observed in maternal samples are consistent with a more established, stable adult nasal community.

The high relative abundance of *Staphylococcus* in both newborn and maternal samples is consistent with its role as a dominant early colonizer of the nasal cavity in adults and infants, with abundance typically declining over the first year of life (Bassis et al., 2014; Teo et al., 2015; Bosch et al., 2017; Liu et al., 2026). By contrast, the maternal nares were enriched for *Corynebacterium* and *Dolosigranulum*, hallmark genera of the healthy adult nasal microbiome that act as stabilizing colonizers and may promote resistance against respiratory pathogens (Biesbroek et al., 2014; Teo et al., 2015; Kumpitsch et al., 2019). *Corynebacterium* remained significantly more abundant in paired maternal samples, and higher levels of both genera have been linked to reduced airway inflammation and lower risk of respiratory infection in children (Biesbroek et al., 2014; Reine et al., 2026). Because *Corynebacterium* has been reported as a dominant taxon transmitted from the maternal to newborn nares, the maternal abundance of these protective genera may help shape subsequent respiratory disease risk in infancy.

Increased nasal *Lactobacillus* has been associated with reduced risk of childhood wheezing illness, and mode of delivery has been proposed as an important determinant of early nasal colonization (Rosas-Salazar et al., 2018). The human vaginal microbiome is dominated by *Lactobacillus* suggesting vaginal delivery may aid in neonatal nares colonization (Miller et al., 2016). Here, we observed higher mean relative abundance of *Lactobacillus* on nasal swabs of newborns delivered vaginally compared to cesarean. Similarly, increased populations of *Lactobacillus* have been noted in the gut microbiome of infants delivered vaginally compared to cesarean (Princisval et al., 2021). We also detected prominent proportions of *Burkholderia-Caballeronia-Paraburkholderia* and *Acinetobacter* in newborn samples. Importantly, our decontam analysis showed that *Burkholderia-Caballeronia-Paraburkholderia*, a recognized reagent contaminant in other low-biomass microbiome studies, was not identified as a contaminant in our dataset. We therefore interpret its elevated abundance in newborn samples as reflecting the sampled community. Indeed, these environmental organisms are commonly found in soil, water, and healthcare settings, and their enrichment may reflect exposure to the delivery-room environment at or shortly after birth (Choi et al., 2012; Dobritsa et al., 2017). The lack of significant difference observed between within-dyad and between-dyad newborn-maternal weighted UniFrac distances, indicate that shared environmental exposure, rather than direct vertical transmission from the mother specifically, may play a more prominent role in early nasal colonization in this cohort.

Because PICRUSt2 infers metabolic potential from taxonomic composition rather than measuring gene content or gene activity directly, predicted pathway enrichments are most informative when interpreted cautiously and in relation to specific community members. The neonatal community was enriched for predicted degradation of aromatic and xenobiotic compounds, including polycyclic aromatic hydrocarbon, fluorobenzoate, furfural, and dioxin. This catabolic potential occurs alongside the elevated abundance of *Burkholderia-Caballeronia-Paraburkholderia* and *Acinetobacter* in newborn samples, two *Proteobacteria* with broad capacity to degrade aromatic hydrocarbons and other recalcitrant compounds (Zhao and Wong, 2009; Morya et al., 2020; Premnath et al., 2021). These predicted functions may represent metabolic traits introduced into the neonatal nares by environmental colonizers acquired around delivery.

A second distinguishing feature of the newborn nasal microbiome was the predicted enrichment of biosynthetic pathways for antimicrobial secondary metabolites, including penicillin and cephalosporin (β-lactam), alkaloid, terpenoid, and polyketide biosynthesis. These observations are notable given the vulnerability of neonates to respiratory pathogen colonization, but they should be interpreted as predicted biosynthetic capacities rather than measured metabolite production (Kemp, 1984; Miller, 2002; Cushnie et al., 2014; Yamaguchi, 2022; Hoffmann et al., 2024). Polyketides, in particular, are valued for broad antimicrobial activity (Kumar and Chopra, 2025), and *Burkholderia-Caballeronia-Paraburkholderia* included species known to produce structurally diverse polyketides (Chen et al., 2019). Such antagonistic interactions have been most thoroughly characterized in the gut, where secondary-metabolite production contributes to colonization resistance (Garcia-Bayona and Comstock, 2018). These data suggest that the neonatal nasal community may be metabolically primed for competitive interactions during the open colonization window immediately after birth, a hypothesis that will require metagenomic and metabolomic validation.

Predicted lipid metabolism pathways, including steroid biosynthesis as well as glycosphingolipid and sphingolipid metabolism, were likewise enriched in newborn samples and occurred alongside the elevated abundance of the lipid-metabolizing taxa *Lactobacillus*, *Acinetobacter*, and *Streptococcus* (Wang et al., 2021; Ren and Palmer, 2023). A plausible substrate source is the newborn’s recent exposure to amniotic fluid and vernix caseosa, both of which are lipid-rich (Checa et al., 2015; Cao et al., 2020). These transient lipids may provide an energy source unavailable in the maternal cavity, supporting the diverse and dynamic colonization observed at birth until the substrates are depleted.

Finally, functional pathways predicted to be enriched in newborn nasal microbiome included active carbohydrate, lipid, and amino-acid metabolism, including sucrose degradation, colonic acid building block biosynthesis, lipopolysaccharide biosynthesis, and adenosylcobalamin (vitamin B12) salvage. These fermentative and biosynthetic capacities are characteristic of lactic acid bacteria and occur alongside a marked enrichment of *Lactobacillus* in the neonatal nares, reinforcing that vertically and environmentally acquired taxa shape not only the taxonomic but also the metabolic distinctiveness of the newborn nasal community in the first hours of life (Pokusaeva et al., 2011; Tang et al., 2023).

Several limitations warrant consideration. First, the relatively modest sample size of 25 maternal and 34 newborn nasal swabs may limit generalizability to broader populations. Future studies incorporating larger and more diverse cohorts will be necessary to validate these observations. Second, the limited number of paired maternal-newborn samples (n=22) reduced our ability to control for participant-level variability, which is substantial in nasal microbiome studies (Biswas et al., 2015). Expanding the number of paired dyads would strengthen comparisons of maternal and neonatal communities. Finally, this study relied on 16S rRNA gene sequencing and inferred functional potential using PICRUSt2 rather than direct measurement of gene content or metabolic activity. Although informative, this approach does not provide the strain-level resolution, direct gene content, or measured metabolic outputs afforded by shotgun metagenomics and metabolomics.

Taken together, these findings reveal that the neonatal nasal cavity is not a sparsely colonized niche at birth but instead hosts a compositionally diverse and functionally versatile microbial community within 24 hours of delivery. This early community appears to reflect contributions from both vertically transmitted and environmentally acquired taxa and is distinct from the maternal nares in both membership and predicted metabolic capacity. By capturing the nasal microbiome at its earliest stage of establishment, our data underscore the perinatal period as a critical window for nasal microbial colonization, with potential consequences for the trajectory of respiratory health. Defining how this initial community matures, and whether its predicted functional potential is realized, will be an important direction for future studies integrating metagenomic, metabolomic, and longitudinal clinical data.

## Data Availability

The sequencing data generated for this study have been deposited in the NCBI Sequence Read Archive under accession number PRJNA1480837.

## Supplementary material

Supplementary Figure 1 **(A)** Rarefaction curves displaying the observed features, Shannon diversity, and sample numbers at various sequencing depths for maternal and newborn samples. **(B)** Bar plots of selected bacterial genera identified by LEfSe plotted as % relative abundance with error bars representing the SEM. Significance between subject groups was measured with a Mann-Whitney test following outlier removal via ROUT (Q = 1). **(C)** Principal coordinate analysis (PCoA) of weighted UniFrac distances comparing the nasal microbiota of female and male newborn. The contribution of subject group to observed variance was determined by permutational multivariate analysis of variance (PERMANOVA).
