# Supplementary material for: The neonatal and maternal nasal microbiomes are compositionally diverse and functionally distinct within 24 hours of birth
Source: Front Cell Infect Microbiol. 2026 Sep 1;16:1922369. doi: 10.3389/fcimb.2026.1922369 (PMC13574624; doi:10.3389/fcimb.2026.1922369)
Supplement: Supplementary file 1 [file Image1.pdf]

Sup. Figure 1.

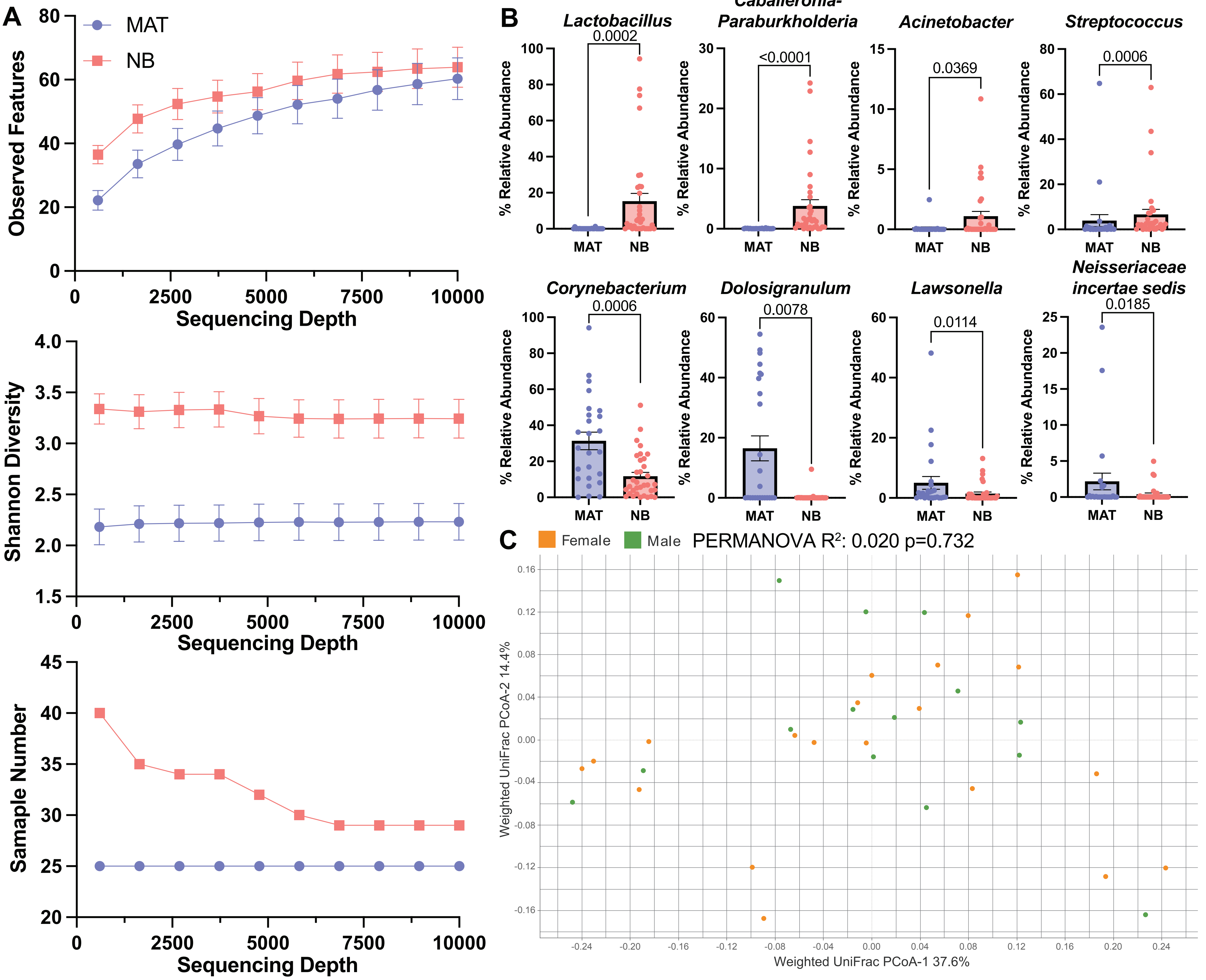
A
MAT
NB
Observed Features
Sequencing Depth
Shannon Diversity
Sequencing Depth
Samaple Number
Sequencing Depth
B
Lactobacillus
0.0002
Burkholderia-Caballeronia-Paraburkholderia
<0.0001
Acinetobacter
0.0369
Streptococcus
0.0006
Corynebacterium
0.0006
Dolosigranulum
0.0078
Lawsonella
0.0114
Neisseriaceae incertae sedis
0.0185
% Relative Abundance
MAT
NB
C
Female
Male
PERMANOVA R^2: 0.020 p=0.732
Weighted UniFrac PCoA-2 14.4%
Weighted UniFrac PCoA-1 37.6%
